# The Challenge of Bringing iPSCs to the Patient

**DOI:** 10.3390/ijms20246305

**Published:** 2019-12-13

**Authors:** María del Carmen Ortuño-Costela, Victoria Cerrada, Marta García-López, M. Esther Gallardo

**Affiliations:** 1 Departamento de Bioquímica, Facultad de Medicina, Universidad Autónoma de Madrid, Spain. Instituto de Investigaciones Biomédicas “Alberto Sols”, (UAM-CSIC), 28029 Madrid, Spain; mcortuno.imas12@h12o.es; 2 Grupo de Investigación Traslacional con células iPS, Instituto de Investigación Sanitaria Hospital 12 de Octubre (i+12), 28041 Madrid, Spain; vcerralopez.imas12@h12o.es (V.C.); martagl.imas12@h12o.es (M.G.-L.); 3 Centro de Investigación Biomédica en Red (CIBERER), 28029 Madrid, Spain

**Keywords:** iPSCs, induced pluripotent stem cells, clinics, clinical trial, drug screening, personalized medicine, regenerative medicine

## Abstract

The implementation of induced pluripotent stem cells (iPSCs) in biomedical research more than a decade ago, resulted in a huge leap forward in the highly promising area of personalized medicine. Nowadays, we are even closer to the patient than ever. To date, there are multiple examples of iPSCs applications in clinical trials and drug screening. However, there are still many obstacles to overcome. In this review, we will focus our attention on the advantages of implementing induced pluripotent stem cells technology into the clinics but also commenting on all the current drawbacks that could hinder this promising path towards the patient.

## 1. The Evolution of a Revolution

In 2006, a team of scientists headed by Dr. Shinya Yamanaka reported one of the greatest breakthroughs in stem cell research: the reprogramming of mouse somatic cells into induced pluripotent stem cells (iPSCs) [1]. Only one year later, in 2007, this challenge was also accomplished in humans by two independent groups [2,3]. Since then, the hope placed in iPSCs-based therapies in personalized medicine has increased considerably, and many groups are trying to take advantage of this new and promising technology.

Due to their self-renewal capacity and their potential to differentiate into any cell type of the three germ layers (endoderm, mesoderm, and ectoderm), iPSCs have been postulated as potential substitutes for embryonic stem cells (ESCs). This is essentially because iPSCs circumvent all the ethical concerns that surround ESCs, the gold standard of human stem cell lines until now. Moreover, the molecular and functional similarity between iPSCs and ESCs is well established [4]. For all these reasons, iPSCs currently represent a great promise in personalized cell therapies, disease modeling, and drug development.

**Figure 1:**
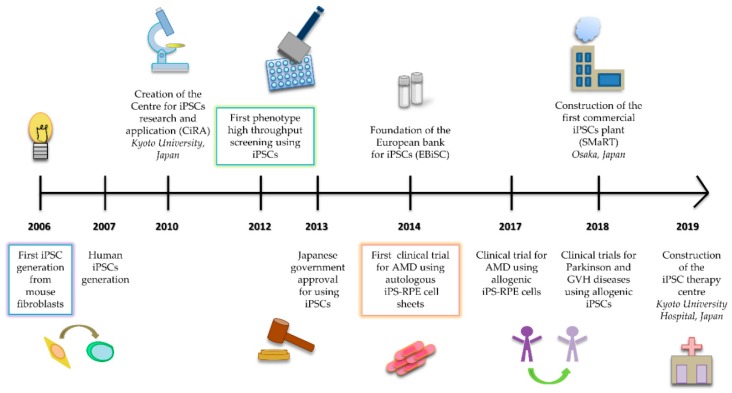
Timeline of the most important milestones from the discovery of induced pluripotent stem cells (iPSCs) in 2006 to now in 2019.

Nowadays, more than a decade after their discovery, the iPSCs technology has evolved rapidly (Figure 1). The scientific community is joining efforts to solve the issues inherent to the use of iPSCs for cell therapy applications, such as their immunogenicity, the risk of teratoma formation, the genomic instability, or the variability among iPSC clones derived from the same donor cells [5].

Although the translation to the clinics may seem easy, this is a very long and complex process with some hurdles that must be still overcome [6]. Notwithstanding these obstacles, as we will see here within, the step from bench to bedside is closer to becoming a reality rather than an unreachable dream.

In this review, we summarize the main applications of iPSCs in the clinics, the progress achieved to date, and the path towards the patient, including the possible drawbacks that might appear in this process.

## 2. The Giant Leap from the Lab to the Clinics

From a practical perspective, iPSCs could be translated to the clinics in two different manners. On the one hand, they could be indirectly used as a platform to produce cell derivatives, such as platelets, or even to develop new drugs. On the other hand, they could be directly employed in regenerative medicine, as an ideal source for cell therapy or even whole tissue transplantation.

In the following sections, we will delve into the indirect and direct uses of iPSCs in the clinics, highlighting some interesting examples and concluding with an overview of the latest clinical trials.

### 2.1. Indirect Uses of iPSCs: a Helpful Tool for the Patient

#### 2.1.1. Platelet Factory

During recent years the number of performed transfusions has significantly increased, mainly due to the upsurge of hematologic diseases. Consequently, to supply the blood demand in hospitals or transfusion centers, the development of new sources of non-immunogenic blood cells is urgently needed. One of the most required cell types is platelets. These are widely used in thrombocytopenic patients, chemotherapy receptors, complex surgeries, etc., but they are not always available due to their short shelf-life, their difficult storage, and the scarcity of donations [7]. In this context, the scalable ex vivo production of iPSCs-derived platelets could solve these technical limitations, as well as the allogeneic problems related to the transfusions [8,9].

Despite the natural safety of platelets conferred by their lack of nucleus, platelet refractoriness is one of the main problems in severe alloimmunized patients who have been transfused on multiple occasions with platelets from donors with different human leukocyte antigen (HLA). This has been overcome by gene editing with CRISPR-Cas9 to generate compatible platelets from iPSCs-derived megakaryocytes [10]. Other approaches have included to knockdown a certain HLA locus, to correct it according to the host or even to generate an HLA-universal iPSC line [11,12].

In addition to the generation of universal platelets, the upscaling of this process is an important consideration before introducing it into the clinics. In vitro platelet production (0.5 platelets/megakaryocyte) is significantly lower than in vivo megakaryopoiesis (>1000 platelets/megakaryocyte) [13]. To improve this ratio, the development of bioreactors has been crucial to yield scalable quantities of functional platelets derived from iPSCs-megakaryocytes. Based on in vivo observations, turbulences are important regulators of thrombopoiesis. For that reason, turbulent flow-based bioreactors have been designed for the production and release of high yield and quality platelets. Furthermore, the successful transplantation of these iPSCs-derived platelets into two animal models has been demonstrated by hemostatic and circulation tests [14]. At this moment, numerous research groups are turning their efforts towards the refinement of the iPSCs-derived platelet production in terms of platelet generation techniques and bioreactor designing. Indeed, this application of iPSCs is actually leading the regenerative medicine race.

#### 2.1.2. The New Age of Drug Discovery

Traditionally, drug discovery and development have been accomplished by mimicking human diseases in animal models, heterologous expression systems, or immortalized cell lines. Over the years, these resources have been very valuable in the pharmaceutical industry. However, when candidate compounds identified using these models are tested in clinical trials, they usually lead to unexpected results. For example, these compounds could cause cardiotoxicity, safety problems, or other undesirable consequences. This is probably due to the limitations of these models to completely mimic the human disease phenotype. In addition, the high inherent costs and the decline in productivity make it necessary to adapt the drug development process to the paradigm shift and develop new procedures in the drug research area.

The capacity to differentiate into many disease-relevant cell types, the availability, and quick evolution of the technology has empowered iPSCs as the perfect tool for the generation of patient-specific cellular models and the so-called personalized medicine. Patient-derived iPSCs could better address specific disease characteristics and sum up the genetic background of the patient. Therefore, iPSCs may be very useful in understanding the interaction between genotype, phenotype, and drug response [15].

In terms of the discovery of novel therapeutic compounds, iPSCs represent a very suitable and almost endless source of cells to perform high throughput drug screenings to identify new drugs among large chemical libraries. This platform also opens up the possibility to carry out drug repurposing studies, i.e., finding new medical uses for existing drugs. As an example, the anti-epileptic drug ezogabine was found to be effective in an iPSC model of amyotrophic lateral sclerosis (ALS), and a clinical trial is currently ongoing [16]. In this study, iPSC-derived motor neurons from ALS patients not only displayed the characteristic disease phenotype (hyperexcitability, increased spontaneous action potentials, and reduced survival) but also showed reduced neuronal excitability and improved cell survival after treatment with ezogabine. Another example of iPSC-based drug screening was accomplished in a patient-derived iPSC model of dystroglycanopathies [17]. This group of congenital muscular dystrophies, often associated with brain malformations, are caused by defective glycosylation of α-dystroglycan. In this case, Kim et al. generated, for the first time, an iPSC line from a dystroglycanopathy patient with severe central nervous system abnormalities. Once they verified that the iPSCs recapitulated the pathological hallmarks of the disease, they successfully differentiated them into cortical neurons. Afterward, 31,954 compounds were screened and, among all of them, one was identified and validated as a potential candidate to treat this disease.

Recently, there has been a resurging interest in phenotypic drug screening, which could complement the actual target-based assays. The former relies on the identification of small molecules, peptides, or interference RNA that might modify the phenotype of the cells in the desired manner [18], whereas the latter measures the effect of compounds on a purified target protein using in vitro assays [19]. In the latest years, a significant percentage of the drugs discovered and validated were found using target-based assays, and, in fact, the traditional phenotypic drug screening was left aside [20]. As mentioned before, one great advantage of iPSCs is that they can better mimic the disease phenotype, covering the whole patient background. That might be the reason why the drug industry is now paying special attention to the retrieval of phenotypic drug screening, despite the great results of target-based assays. Therefore, a combination of empirical (phenotypic) and molecular (target) approaches would be the ideal challenge for a successful drug screening [21]. One of the first studies in phenotypic drug screening was published by Lee et al. in 2012. They reported the identification of new compounds in familial dysautonomia-iPSCs by phenotypic high throughput screening [22]. This rare genetic disorder entails a marked reduction in the IκB kinase complex–associated protein (IKAP), caused by mutations in the *IKBKAP* gene. Their first hit was the identification of multiple disease-specific phenotypes by the generation of several iPSCs lines from patients with dysautonomia. After that, a primary screen of 6912 small molecules was accomplished in the iPSC-derived neural crest progenitors, performing fold-difference analysis in the gene expression. Finally, eight compounds that significantly rescued IKAP expression were found. Several signs of progress have also been made in the knowledge of fibrodysplasia ossificans progressiva (FOP) by modeling the phenotypes with patient-derived iPSCs [23]. In this case, the correction of the causal mutation of FOP in the generated iPSC lines was crucial to elucidate the key genes associated with FOP onset and their role during chondrogenesis. Moreover, rapamycin has been postulated as a promising therapeutic compound to prevent the development of ossification in patients.

The fact that iPSCs can be derived from the patient and mimic the biochemical or metabolic aspects of the disease could be very useful to probe the bioactivity and toxicity of the drug in the early clinical stages of drug development. Actually, there are some studies focused on comparing the drug effect concurrently in patients and their iPSCs [24]. Moreover, pre-clinical safety and efficacy tests could be performed on patient-derived iPSCs, control iPSCs, and corrected iPSCs from patients to find potential side-effects. In that sense, the generation of isogenic iPSC lines with the correction of the disease-causing mutation could solve the problems related to the genetic background variance [25]. In 2018, Japanese researchers revealed ropinirole hydrochloride as a potential candidate drug for ALS treatment. They generated spinal motor neurons derived from patient-iPSCs and performed, successfully, the hit validation. At the present time, this promising study is in phase I/IIa, with tolerability and efficacy of the aforementioned drug also being explored using patient-derived iPSCs. This illustrates well how iPSCs have joined the drug discovery field [26].

The latest trend in iPSCs and drug discovery is the organ-on-a-chip (OOC) technology. These are micro-devices that combine microfluidics, iPSCs, and tissue engineering to generate three-dimensional self-organizing tissues. These platforms aim to replicate the physiological characteristics of tissues and organs of a certain group of patients or individuals to predict human global responses to drug treatments [27]. Several OOCs examples of different organs (liver, lung, heart, intestine, or blood–brain barrier) have been constructed and are now a powerful tool for the study of drug pharmacodynamics, pharmacokinetics, and toxicity profiling [28].

The use of iPSCs in drug screening foreshadows, presumably, a promising near future in the research field and the pharmaceutical industry, shortening the drug development time and reaching a unique goal: finding the maximum benefit with better drugs.

### 2.2. The Potential of iPS Cell-Based Therapeutics

Apart from those applications mentioned previously, it is feasible to use iPSCs in the clinics in a more direct way. Cell therapy turns out to be a relatively novel technique that aims to implant human cells into a patient to repair or replace an injured tissue or restore the functionality of an organ [29]. This is especially useful for organs with poor regenerative capacity, such as the heart, since adult stem cells are unable to unravel the serious problem of tissue damage or disease [30].

In this context, endogenous stem cells have been broadly explored to be applied to these approaches [31]. For example, cell therapeutics with mesenchymal stem cells (MSCs) have proven evidence for efficacy and safety in animal models. This fact, along with the ease of manipulation, has boosted the approval of various clinical trials with MSCs [32]. However, the short lifespan associated with adult stem cells strongly limits their use in the clinics [33]. At the present time, iPSCs are almost certainly the most promising source for cell replacement therapies and tissue engineering. This is mainly due to their self-renewal capacity and the possibility to potentially give rise to any kind of cell [34]. Nevertheless, iPSCs differentiation protocols towards different cell types require vast enhancement of efficiency rates to obtain the proper maturation, as we will discuss later [35]. Once this is accomplished, the application of iPSCs technology could ideally satisfy the demand that cannot be fulfilled only by organ transplantation [36].

iPSCs technology opens a wide range of therapeutic possibilities, employing different strategies for different necessities. Functional cells could be engrafted, either as stem cells or differentiated to another cell type, to replace the damaged ones [37]. It is also possible that injected stem cells secrete growth factors stimulating the repair of non-functional cells [38]. Occasionally, the use of iPSC-derivatives for regenerative medicine is limited by the low therapeutic efficacy of transplanting cells alone, as their survival and differentiation potentials may be compromised [39]. Thus, tissue engineering emerges as the combination of cells with the optimal biomaterials for achieving organ or tissue regeneration. The development of a suitable scaffold to support cells in vivo could ameliorate cell viability and their structural stability, guiding the growth of the new tissue [40].

In stem cell therapy, autologous transplantation would be idyllic to overcome the complications associated with immune rejection. The likelihood of generating iPSCs from the patient’s somatic cells has revolutionized the field, considering that these iPSCs could possibly be differentiated into the cells affected in the particular disease and eventually transplanted back into the patient [41]. In this scenario, and thanks to the great advances in genome editing technologies, personalized medicine is now on the horizon. Disease-causing mutations could be precisely corrected in patient-derived iPSC lines as a means to achieve more specific clinical treatments [30]. The main problem associated with autologous transplants is that they imply the necessity to carry out the whole extensive procedure for each individual. The process of somatic cell reprogramming and iPS cell differentiation is very time-consuming and present technical difficulties, without contemplating the elevated costs [42].

On the other side, allogeneic transplantation is associated with the concomitant requirement of administrating immunosuppressive drugs. Although long-term administration appears to be indispensable in most cases for preventing rejection, the immunosuppression degree needs to be determined for each concrete engraftment [43]. To solve these issues, another possibility would be the allogeneic transplantation of iPSCs obtained from a donor who is HLA compatible with the patient, which would reduce immune rejection [44]. In this regard, the feasibility of creating a genomic stability-validated iPSC bank containing homozygous cell lines to allow the HLA matching for a large number of potential recipients is being widely explored. Those biobanks would enable fast access to a high amount of cells, which is actually the main purpose [45].

### 2.3. Preclinical Studies and Ongoing Clinical Trials

It is important to highlight that translation into the clinics still entails a long road ahead. In the preclinical stage, the therapeutic response has to be predicted. In this sense, animal modeling is a highly valuable tool to foresee if grafted cells could integrate and fulfill their function correctly [46]. After that, the different clinical trial phases assure that essential parameters, such as tumorigenicity, dose toxicity, and immunogenicity, are assessed before finally approving the product to be grafted into the patients [44].

At the present time, iPSC technologies have reached a point where several cellular therapies have started their way towards the clinics [43]. In this section, we will focus on the main achievements in iPSC-based therapies, going through the development of every remarkable clinical trial (Table 1).

To date, the more notable progress has been made for retinal degeneration diseases, specifically for age-related macular degeneration (AMD). In 2009, preclinical data demonstrated for the first time the recovery of visual function when injecting retinal pigment epithelium (RPE) differentiated from iPSCs in a rat model’s retina [56]. A great leap forward was taken when the group headed by Masayo Takahashi at the Riken Centre for Developmental Biology in Japan generated iPSC-RPE cell sheets in 2014. They characterized them to meet clinical use requirements and reported successful transplantation in a primate model, without any complications [57].

These events led to the initiation of the first iPSCs clinical trial in the same year. Investigators at the Riken Centre grafted an autologous iPSC-RPE cell sheet under the affected retina, without immunosuppression, in a 77-year-old woman with AMD [47]. However, the trial was suspended the following year, due to the detection of a genetic copy-number alteration in the iPSCs of the second patient who was enrolled [58]. One year post-transplantation, the progression of the degeneration was halted, an area with photoreceptors recovery was observed, and her vision remained stable [47,59]. There were no signs of immune rejection or tumor formation. Hence, they concluded that the transplantation was safe [60]. However, it was doubted if the sheet was tolerated because of the eye’s immunoprivileged nature and whether this would be ever accomplished in other organs [61]. Recently, investigators involved in this clinical trial have shifted their approach toward the use of HLA-matched allogeneic iPSCs. After a successful proof-of-concept in non-human primates [62], in March 2017, they announced that a 60-year-old man was the first patient to receive iPSC-RPE cells derived from another person [48]. Furthermore, a clinical-grade iPSC bank from healthy HLA homozygous donors is now being established at the Centre for iPS Cell Research and Application (CiRA) in Kyoto (Japan) [63]. The latest advance has only recently taken place in July 2019, when Kohji Nishida and colleagues at Osaka University initiated a clinical trial for limbal stem cell deficiency, a condition in which corneal stem cells are lost. They grafted a sheet of iPS-derived corneal cells into the cornea of a patient, and just one month later, her vision seemed to have improved [49].

Neurological disorders have likewise attracted a lot of attention, with therapies for Parkinson’s disease clearly being the most advanced. Since Wernig et al. confirmed in 2008 that symptoms and dopaminergic function of rat models improved when injecting iPSC-derived dopaminergic neurons [64], significant progress has been made in this sector. Just a few years ago, in Japan, dopaminergic progenitor cells differentiated from iPSCs were transplanted into a primate model of Parkinson’s disease and showed to proper function [65]. Moreover, dopamine precursor cells were derived from iPSCs established out of HLA-homozygous healthy individuals by Jun Takahashi’s research group at Kyoto University. In October 2018, the second clinical trial using iPSCs started when these precursors were implanted into the brain of a patient in his 50 s, amongst a total of seven patients recruited [50].

Furthermore, iPSCs-derived products are also highly valuable in immunotherapy for patients with solid tumors. In 2016, the administration of iPSC-derived natural killer (NK) cells into a mouse model provided proof of their potential for effective immunotherapy treatment of ovarian cancer [66]. Quite recently, in February 2019, allogeneic iPSC-derived NK cells generated by a research group at the University of Minnesota, in collaboration with Fate Therapeutics, were approved for a clinical trial. Their aim is to treat up to 64 patients with diverse cancer types using either monotherapy alone or in combination with immune inhibitors [51]. In addition, iPSC-based therapies are being conducted for graft-versus-host disease (GVHD). The Australian company Cynata Therapeutics received approval to proceed with a clinical trial of allogeneic iPSC-derived mesenchymal stem cells (MSCs). They established Cymerus^TM^ iPSC-MSCs and tested preclinical efficacy in a humanized mouse model [67]. In 2018, phase I of the trial was completed in 16 steroid-resistant GVHD patients with positive results, supporting progression to a phase II trial [52].

Following these steps, other diseases have a prospective therapy in their developmental path. For spinal cord injuries, preclinical studies with iPSCs-neural progenitor cells in a non-human primate model provided evidence for remyelination and locomotor function recovery [68]. In February 2018, the Japan government gave the go-ahead to Professor Hideyuki Okano for a clinical trial aimed at treating patients with spinal cord injuries at Keio University (Tokyo) [53,69]. Conversely, in 2017, iPSC-derived cardiomyocytes were transplanted by Kawamura et al. in a porcine model of ischemic cardiomyopathy using a cell-sheet technique. Cardiac function was markedly improved, and neovasculogenesis was stimulated [70]. Recently, Yoshiki Sawa and colleagues at Osaka University have attained the approval for a clinical trial to graft allogeneic sheets of tissue derived from iPSCs onto diseased hearts of three patients [54]. Likewise, a group from Kyoto University has obtained the endorsement to begin a transfusion trial using platelets derived from iPSCs of an individual with aplastic anemia [55].

In addition to all the clinical trials performed up to now, preclinical studies are also being explored for other diseases [71]. Clearly, iPS-based cellular therapies have progressed largely, considering that only a few years ago, iPSCs had not been tested in clinics, and, now, clinical trials are increasingly emerging.

## 3. Where We Are and Where We Are Heading to

It is absolutely without doubt that the emergence of iPSCs technology marked a turning point in biomedical research [72]. The ten-year anniversary of the iPSCs breakthrough in the scientific landscape was celebrated in 2016. This was a point where many researchers reflected if the initial enthusiasm about all the potential of these cells had only been an illusion, or if they could actually take the leap from the culture dish to the patient.

**Figure 2:**
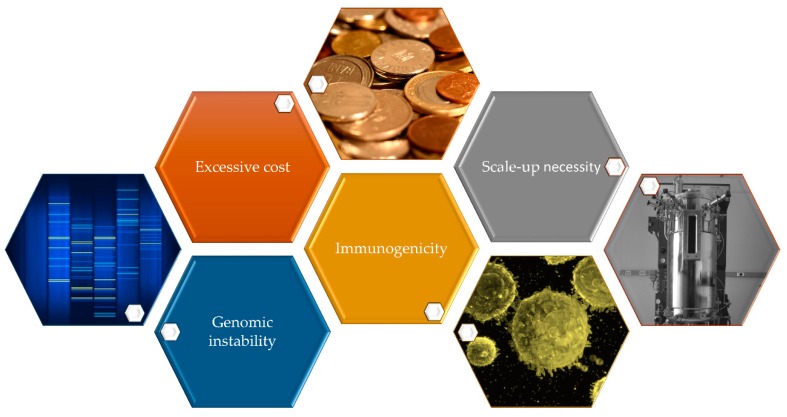
Principal hurdles encountered when translating iPSCs to the clinics.

The advantages of iPSCs are extremely well-known at many distinct levels, for instance, their capacity to differentiate to any cell type or even their potential to create a patient-derived disease model [73]. However, there are many barriers to encounter that, unfortunately, nowadays block their way to the patient. After reviewing some of the main applications of iPSCs in the clinics, we will now discuss the principal drawbacks which are trying to be surpassed in the path towards a clinical translation (Figure 2).

### 3.1. Economic Issues

First, iPSC technology is rather costly. It is estimated that the generation and expansion of an iPSC line, along with all the necessary tests to assess its pluripotency and safety, cost between 10–20,000 US dollars and require between 4 and 6 months for its production [74]. However, when preparing the cell line for the clinics, these costs can reach up to 1 million dollars. It is reasonable to think that there is an urgent need to find a cost-effective manner to overcome this hurdle, which would definitely ease the iPSCs translation to the clinics. In this regard, although autologous therapies can be particularly attractive in terms of personalized treatments [75], the current cost of such therapies would indeed be prohibitive and definitely time-consuming [76]. That is why allogeneic therapies may be a good alternative, as they are more economical and could reach a wider number of patients. Nonetheless, several additional difficulties exist which could hold up their immediate application.

### 3.2. Genomic Instability, an Old Enemy

One of the main concerns that arose soon after the development of iPSCs was their genomic stability. Chromosomal aberrations are quite common during the reprogramming process, and other types of mutations have been found at a significant frequency [77]. In fact, as presented before, the first clinical trial involving iPSCs-derivatives was suspended because of the identification of copy number alterations in the second patient’s iPSCs, which were not found in the primary fibroblasts [78].

Mutations present in iPSCs can arise from three different origins: they can be induced by the subsequent passages of these cells, induced by the reprogramming process itself, or they can be mutations already existing in the primary cells [79]. However, the principal issue is the consequences of those mutations, as some of them could lead to an increased tumorigenic potential. It is necessary to address this safety issue for iPSCs in clinical use and optimize the reprogramming and manufacturing conditions of these cells in a very strict manner to ensure the possibility of a derived therapy.

### 3.3. Immunogenicity: the Main Obstacle?

The announcement in 2011 that iPSC-derived teratomas could elicit a T-dependent immune response in syngeneic mice soon derived in a general skepticism about the therapeutic options for these cells, as even autologous iPSCs seemed to be immunogenic [80]. After that, many groups started to investigate the immunogenicity of iPSCs, since if it was confirmed that they could trigger an immune response in the host, their possible applications in the clinics would be ruled out immediately. However, several posterior studies started to question this statement, indicating that iPSC-derived cells are not immunogenic by themselves and are well tolerated by the immune system [81,82]. Subsequently, Zhao and co-workers utilized a humanized mouse model to test the immunogenicity of several distinct iPSC-derived cells, finding that the immune response was different from one kind of derivative to another [83]. Thus, the immune response elicited by iPSCs or their derivatives seems different depending on the cell line in question. This response may be triggered by epigenetic alterations present in the cell lines or even genomic abnormalities that give rise to aberrant immunogenic products [84]. Although the complete lack of immunosuppressive drugs in iPSC-derived therapies maybe a difficult goal to reach, the deep understanding of the immune response occasioned by these cells can be a huge step forward towards greater safety and a decrease in the necessity of immunosuppressive treatments in the case of iPSCs transplants [85].

### 3.4. Biobanking the iPSCs

As reviewed earlier, the cost and time required for autologous therapies to become a reality for the patients are, unfortunately, two of the main reasons why they seem to be far from the clinics [35]. Perhaps the alternative of allogeneic transplants could be the optimal solution, but the problems associated with immunogenicity are the main drawback. In theory, there could be three different scenarios in which the immune rejection could be minimal: whether the iPSCs are remaining for a short period inside the patient, whether they are not immunogenic by themselves, or whether they are to be injected into an immune-privileged site [86]. However, in many cases, the immunogenicity cannot be excluded from the equation, constituting one of the main problems criticized in the clinical translation of iPSCs. For this reason, many researchers began proposing the possibility of creating an iPSCs biobank characterized in terms of the HLA, to not only reduce the costs of the technology itself but to minimize the immune rejection problem and facilitate the possibility of bringing the iPSCs therapy to reality [45].

As the HLA system is highly polymorphic, it has been suggested that considering only the loci HLA-A, HLA-B, and HLA-DR would be sufficient to reduce the rejection and the doses of immunosuppression required. The proposal would be to create an iPSCs bank from HLA-homozygous and blood group O donors, simplifying the patient-donor matching [87]. In fact, it has been estimated that 150 HLA-homozygous iPSC lines would be sufficient to match 93% of United Kingdom recipients [88], and 140 selected donors would be enough for the 90% of Japan population [89]. Maybe the creation of several biobanks with selected lines considering the HLA diversity on different populations, could cover the majority of the iPSC lines required worldwide. Additionally, it is pivotal to remark that HLA-characterized iPSCs biobanks for a clinical application would also need specific requirements, standardized manufacturing protocols, and safety controls much stricter than for research to ensure the security of the potential treatments developed [90].

### 3.5. Loose Ends in the Clinical iPSCs Application

The application of iPSCs in the clinics seems to have many ups and downs. The huge potential of this technology is absolutely unquestionable, but the blockade due to some aspects mentioned previously still remain to be resolved. Moreover, the standardization of the reprogramming protocols and the implementation of good manufacturing procedures for the manipulation of these cells seem crucial to obtain a good translational product [91].

Currently, the production of iPSCs is still restricted to the laboratory, which greatly limits their applicability. It would be necessary to adjust the conditions and methods for a large-scale expansion of the iPSCs production, mainly since cellular therapies would need large quantities of them to be feasible [92]. The monitoring of the toxicity and tumorigenicity, as well as the assessment of the safety of the cells, is absolutely indispensable for the clinical application of iPSCs [93].

Additionally, there is a present need to optimize the differentiation procedures to derive iPSCs to the specific lineage of interest [94]. Theoretically, the potential of iPSCs is based on their capacity to differentiate virtually to any cell type, but in practice, the reality is rather different. It is frequent that the differentiation gives rise to an undesirable phenotypic heterogeneity and a lack of maturity, with low-efficiency rates [95]. In terms of iPSCs differentiation, there are several distinct approaches that could be implemented. Some protocols are based on the introduction of a certain critical transcriptional factor related to the cell type in question, often with the use of viral vectors [96]. The main problem here would be the integration within the genome, which could compromise the safety of the iPSCs-derivatives. Another kind of protocol is centered on mimicking embryonic development with the addition or elimination of strategic molecules in the cell culture medium, trying to activate or inhibit certain key pathways and distance iPSCs from their pluripotency [97,98]. However, the incapacity of any of these protocols to achieve a complete maturity is the main drawback, especially in certain cell types, such as cardiomyocytes, and diseases where the affected phenotype is only exhibited at a very late stage [99,100]. That is why new approaches are being tested to resolve this issue. For instance, some groups are trying to achieve a more mature differentiation state based on the use of different small molecules cocktails, the manipulation of the cellular growth environment, or even the implementation of 3D approaches [101,102].

### 3.6. A Glimpse of the Future

There are several novel strategies that seek to overcome some of the pitfalls previously mentioned, shedding light on the possibility of widespread utilization of iPSCs in the clinics. For instance, the introduction of a suicide gene based on the herpes simplex virus thymidine kinase could incorporate some sort of “emergency switch” which could be activated by a non-toxic drug when the iPSCs were not needed anymore or in case of a problem, such a tumorigenic risk [103]. Alternatively, Deuse and colleagues have tried to overcome the problem of immunogenicity by inactivating some key components of the major histocompatibility complex (MHC) class I and II with CRISPR/Cas9 technology and overexpressing the transmembrane protein CD47 [104]. This way, they have created hypoimmunogenic cells, which elude the host’s immune response, opening the door to the possibility of creating universal non-immunogenic cells [105]. Although all these new approaches are still at the early stages of development, it does seem clear that the advances in the field are taking place at a high velocity. Therefore, it would not be surprising that the translation of iPSCs to the clinics would continue to grow with more clinical trials and cellular therapies appearing in the near future.

## 4. Concluding Remarks

The potential of iPSCs is not only restricted to basic research in a laboratory as disease models or pathophysiological studies but nowadays, they have already taken the leap from bench to bedside. Cell therapy illustrates this huge advance with several ongoing clinical trials and many more to come. The examples in age-related macular degeneration and Parkinson’s unveil that, after the scientific efforts to develop the technology, and despite their inherent hurdles, iPSCs can successfully treat the patient. In the meantime, iPSCs are already a very powerful tool in the development of new therapeutic drugs, and they can even be used as an intermediate to generate other products of interest as platelets. Nevertheless, many issues related to the clinical employment of iPSCs are still controversial, such as immunogenicity, genome instability, or tumorigenicity. Furthermore, the elevated cost of their production, along with the necessity to standardize the technical protocols, restrain the scaling up of this promising technology, which would be necessary to bring it to a greater number of patients. With this in mind, it is important to note that new strategies are being developed to solve these pitfalls, which will prompt the definitive implementation of this technology on a wider scale.

## Figures and Tables

**Table 1:** Clinical trials for the therapeutic application of induced pluripotent stem cells (iPSC) derivatives.

Disease	Cell Type	Transplant Type	Institution and Country	Study Start Date *	Registration Number	Status	Reference
Age-related macular degeneration	Retinal pigment epithelial cells	Autologous	RIKEN Centre for Developmental Biology (Japan)	October 2013	UMIN000011929	Phase I (suspended)	[47]
Age-related macular degeneration	Retinal pigment epithelial cells	Allogeneic	RIKEN Centre for Developmental Biology (Japan)	Unknown	Unknown	Phase I	[48]
Limbal stem cell deficiency	Corneal stem cells	Allogeneic	Osaka University (Japan)	Unknown	Unknown	Phase I	[49]
Parkinson	Dopamine precursor cells	Allogeneic	Kyoto University (Japan)	July 2018	UMIN000033564	Phase I/II	[50]
Cancer	Natural killer cells	Allogeneic	University of Minnesota (US) and Fate Therapeutics (US)	February 2019	NCT03841110	Phase I	[51]
Graft-versus-host disease	Mesenchymal stem cells	Allogeneic	Cynata Therapeutics (Australia)	October 2016	NCT02923375	Phase I completed	[52]
Spinal cord injury	Neural progenitor cells	Allogeneic	Keio University (Japan)	N/A	N/A	Not yet recruiting	[53]
Heart failure	Cardiomyocytes	Allogeneic	Osaka University (Japan)	June 2018	UMIN000032989	Not yet recruiting	[54]
Aplastic anemia	Platelets	Autologous	Kyoto University (Japan)	N/A	N/A	Not yet recruiting	[55]

* The study start date refers to the date at which the study was registered for the first time.

## Abbreviations

iPSCs	induced pluripotent stem cells
ESCs	embryonic stem cells
HLA	human leukocyte antigen
ALS	amyotrophic lateral sclerosis
FOP	fibrodysplasia ossificans progressiva
IKAP	IκB kinase complex–associated protein
OOC	organ-on-a-chip
MSCs	mesenchymal stem cells
AMD	age-related macular degeneration
RPE	retinal pigment epithelium
CiRA	center for iPS cell research and application
NK	natural killer
GVHD	graft-versus-host disease
MHC	major histocompatibility complex
